# Chemical meningitis in children as a risk factor following craniopharyngioma resection – a case report

**DOI:** 10.1186/s12883-020-01638-y

**Published:** 2020-02-15

**Authors:** Magdalena Chrościńska-Krawczyk, Ewa Zienkiewicz, Arkadiusz Podkowiński, Maria Klatka

**Affiliations:** 1 Department of Paediatric Neurology, Faculty of Paediatrics, Medical University of Lublin, Al. Racławickie 1, 20-059 Lublin, Poland; 2 Department of Paediatric Neurology, University Children’s Hospital in Lublin, Lublin, Poland; 3 Department of Neurosurgery and Paediatric Neurosurgery, Medical University of Lublin, Lublin, Poland; 4 Department of Paediatric Endocrinology and Diabetology, Medical University of Lublin, Lublin, Poland

**Keywords:** Craniopharyngioma resection, Chemical meningitis, Children

## Abstract

**Background:**

Craniopharyngiomas are defined by the WHO as “benign” tumours, but their location and surgical treatment may be associated with major complications, one being chemical meningitis. Although rare, especially in children, it should be taken into account when worrying symptoms appear after surgery.

**Case presentation:**

The aim of this study is to present the case of chemical meningitis in a 7-year-old girl. She was admitted to the Department of Neurology with the following symptoms: headache, vomiting and balance disorders. Brain magnetic resonance imaging showed a tumour in the sellar and suprasellar region, which was diagnosed as a craniopharyngioma. Due to acute hydrocephalus the patient underwent emergency surgery. Conventional surgery was preceded by an endocrinological consultation to determine pituitary hormone levels. The first 6 days post-surgery, during which the patient started substitution therapy for pituitary insufficiency, were uneventful but on the seventh day she presented with seizures, fever, severe headache, weakness, irritability, stiffening of the neck and a gradual degradation of consciousness. This clinical presentation suggested meningitis, which was confirmed by examination of cerebrospinal fluid.

**Conclusions:**

The conventional and/or endoscopic resection of a craniopharyngioma poses a risk of postoperative complications in the form of chemical meningitis. Although this is a rare occurrence in children with craniopharyngioma, physicians should be aware of this complication and its clinical presentation as it may facilitate earlier diagnosis, appropriate treatment and a faster recovery of their patients.

## Background

The first description of postoperative aseptic meningitis was published in 1928 by Cushing and Bailey [1]. The ICD-O 3rd revision references the code number 9350, which refers to “unspecified craniopharyngioma”, and the following codes which correspond to the two histological subtypes, adamantinous (9351) and papillary (9352) craniopharyngiomas [2]. The incidence of newly diagnosed craniopharyngiomas ranges from 0.13 to 2 per 100,000 population per year, with a point prevalence of 1 to 3 per 100,000 population [3].

Aseptic meningitis has been reported after supratentorial surgeries, and seems to be more frequent in patients who undergo operations for non-tumorous intracranial pathologies rather than those with brain tumours [4, 5]. Craniopharyngiomas are classified as benign, however, they should be considered as “malignant” due to the possibility of neurological and endocrine complications occurring. The rate of surgical mortality oscillates around 0.6%, and the overall survival rates at 1 and 5 years are 95 and 90%, respectively. The only negative predictor of survival is progressing age. Patients may suffer from postoperative complications [6].

The literature describes 64 cases of complications after endoscopic endonasal surgery for craniopharyngioma in which 7.8% of patients were diagnosed with postoperative meningitis. However, in this study no distinction was made between infectious and non-infectious aetiology. In another study, where 1146 patients were followed after removal of tumours at the cerebellopontine angle, the overall rate of meningitis was 4.54%, and the rate of aseptic meningitis was 2.61%. These data can indicate that aseptic meningitis represents a significant portion of all postoperative cases [7].

This article aims to 1) present the case of chemical meningitis as a complication of post-surgical removal of a craniopharyngioma and 2) discuss the diagnosis and management of aseptic meningitis. The diagnosis was preceded by: a neurological examination, an endocrine examination, magnetic resonance imaging (MRI), neurosurgical treatment, blood biochemistry and an examination of cerebrospinal fluid (CSF).

Children operated on for a craniopharyngioma may develop chemical (aseptic) meningitis. Such patients require antibiotic therapy despite a negative CSF result.

## Case presentation

**Fig. 1:**
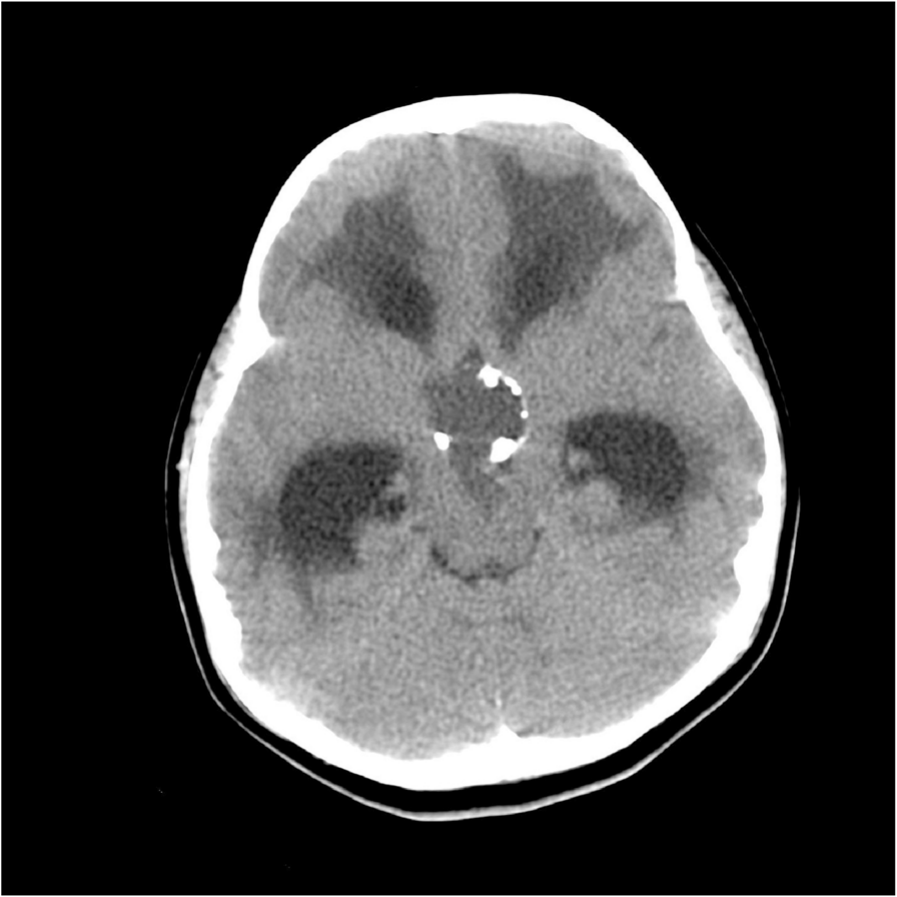
Preoperative CT of the head with contrast (16 June 2016). The tumor, 25x40x31 mm (I-I, a-p, c-c) in size, compresses onto the 3rd ventricle and interventricular foramina. Ventricular system of the brain distended (Evans ratio 44%), symmetrical, with the signs of a transependymal oedema

A 7 year-old girl was admitted to the Neurology Department with severe headache, vomiting, muscle weakness and balance disorders. She had no history of previous disease and her psychomotor development was normal. However, 2–3 months before admission, the child’s behaviour showed problems with concentration. Preoperative computed tomography (CT) revealed the existence of a large cystic tumour with peripheral calcifications expanding from the sella to the third ventricle area and acute hydrocephalus (Fig. 1).

**Fig. 2:**
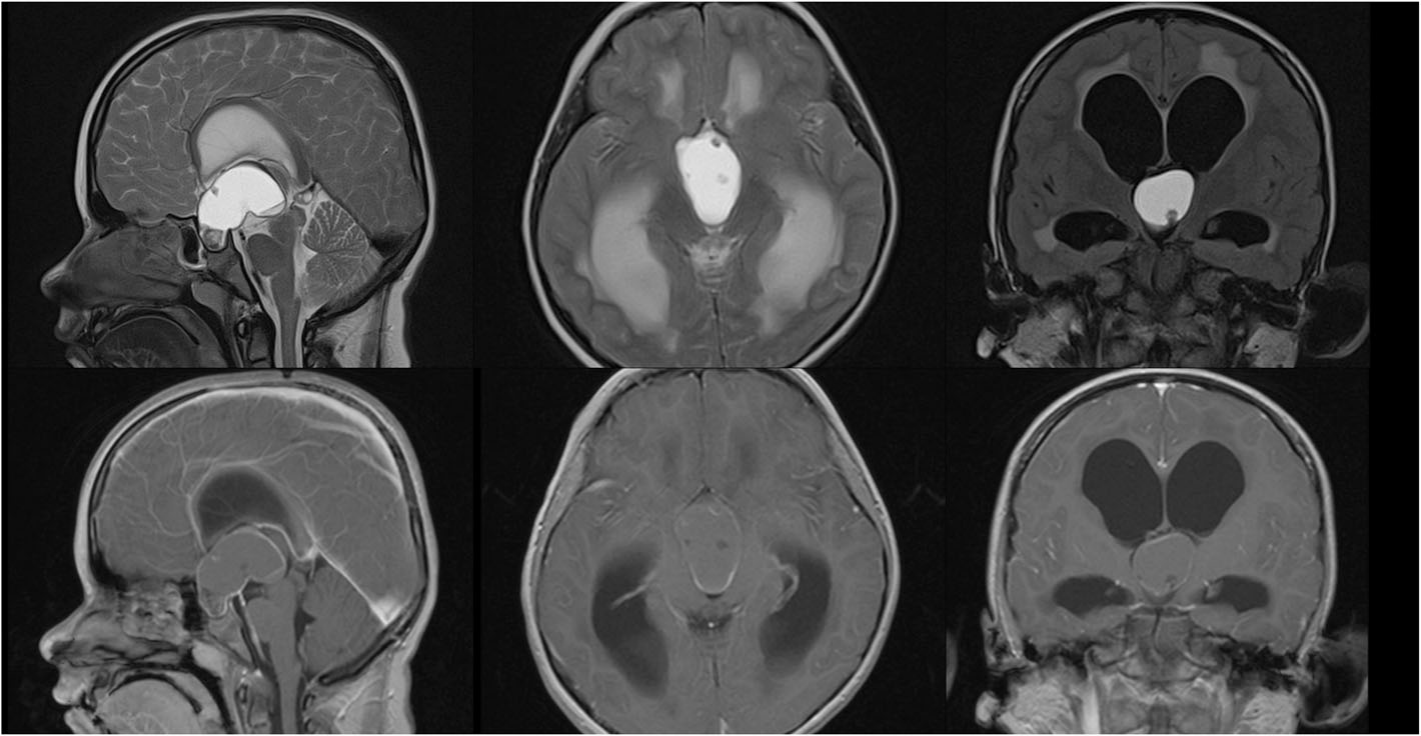
Preoperative magnetic resonance imaging scan (17 June 2016). The pathological area with fluid accumulation (APxCCxLR: 45x35x27 mm) in sellar and suprasellar region. Tumor tissue well demarcated. The suspicion of proliferative process, craniopharyngioma. Supratentorial part of the ventricular system shows characteristic features of hydrocephalus with transependymal oedema

Magnetic resonance imaging identified a large solid pathological area with fluid accumulation in the sellar and suprasellar region (Fig. 2).

Based on these findings, the patient was diagnosed with a tumour expanding from the sella to the third ventricle, most likely an adamantinomatous craniopharyngioma with concomitant acute occlusive hydrocephalus. The recommended treatment included a right-sided pterional craniotomy with extensive resection of the tumour.

A deterioration in the patent’s status necessitated immediate surgery with resection of the tumour. A pterional craniotomy was performed on the right side under general anaesthesia with the patient in a supine position. After reaching the base of the brain and evacuation of fluid from basal cisterns, the tumour’s wall was cut, and a thick yellowish fluid was drained. The tumour was separated from the right optic nerve, right ICA, optic chiasm and left optic nerve. Instead of the pituitary stalk, two thin bands were found, extending from the third ventricle and running towards the sella turcica on both sides of the tumour. After separating the tumour from those bands, it was incised slightly above the sellar diaphragm, dissected from the third ventricle wall, along the lamina terminalis, and removed. After achieving haemostasis, the dura mater was sutured and fixed to the bone and a cranioplasty was performed. A suction drain was then placed, soft tissues were closed in layers and protected with a dressing. Pathomorphological examination of the surgical specimen revealed a craniopharyngioma G1-WHO testing positively for AE1/AE2 (stratified epithelium), GFAP (reactive gliosis), MIB-1 (with a few epithelial and inflammatory cells) and CD 68 (multiple macrophages, including polynuclear ones).

The girl was stable, conscious with good intervention and showed no paresis. She received prophylactic vancomycin, meropenem and fluconazole, fluids for improving water-electrolyte balance, substitution therapy with thyroid and adrenal hormones, and the treatment of diabetes insipidus. The first 6 days after the surgery were without complications. Neurological examination showed no signs of focal damage to the central nervous system. Due to multi-hormonal pituitary insufficiency, substitution therapy was introduced with L-thyroxine, hydrocortisone and desmopressin, adjusting the doses to serum endogenous hormones.

On the seventh day after surgery, the patient’s status rapidly deteriorated. She reported a severe headache and had a fever. The patient became sleepy and apathetic. She periodically loss consciousness and had recurrent seizures lasting approximately 15 s. A neurological examination revealed stiffening of the neck, positive Kernig’s and Babinski signs on the right side, and convergent strabismus in the left eye.

The result of a head CT did not suggest the need for repeat surgery. A lumbar puncture was performed. CSF was fully transparent with 18 cells per μl (normal range: 0–5), 60% of neutrophils and 38% of lymphocytes, a positive Pandy’s reaction, a negative Nonne-Apelt reaction, 154.0 mg/dl of protein (normal range: 15–45), a normal concentration of glucose (79 mg%, normal range: 50–80), 2.7 mmol/l of lactic acid (normal range: 0.5–2.2). A latex test and aerobic and anaerobic cultures of CSF yielded negative results. The patient tested negatively for anti-CMV, anti-EBV, anti-HSV, anti-poxvirus and anti-Borrelia burgdorferi IgM antibodies. The results of other tests, including aerobic and anaerobic cultures of the blood (collected at peak fever) and urine, were also negative. A microbiological examination of throat and nasal swabs revealed a presence of physiological microflora: Neisseria spp. and *Streptococcus viridans*.

Because of a lack of an etiological factor for bacterial or viral meningitis, the patient was diagnosed with aseptic (chemical) meningitis. Antibiotic therapy treatment included vancomycin, ceftriaxone, fluconazole, acyclovir, and nystatin and was maintained for 14 days. An antiepileptic agent, valproic acid, was administered since both clinical presentation and ictal electroencephalographic findings suggested epilepsy. Hormonal treatment was continued. The patient was fed enterally via a catheter with a semiliquid low-sodium diet.

Increasing intracranial pressure required the placement of external drainage on day 11 after tumour resection. On day 14 post-surgery, the external drainage was removed following a CT scan of the head which showed the ventricular system without features of hydrocephalus. After removal of the drain, the patient was in good general status and was verbally responsive. Neurological examination showed the presence of meningeal symptoms and slight strabismus in the left eye, decreased muscle tone and diminished asymmetrical tendineae reflexes in the upper extremities, with a predominance of the left side, and a decrease in muscle tone with areflexia and a positive Babinski sign on the right side in the lower extremities.

**Fig. 3:**
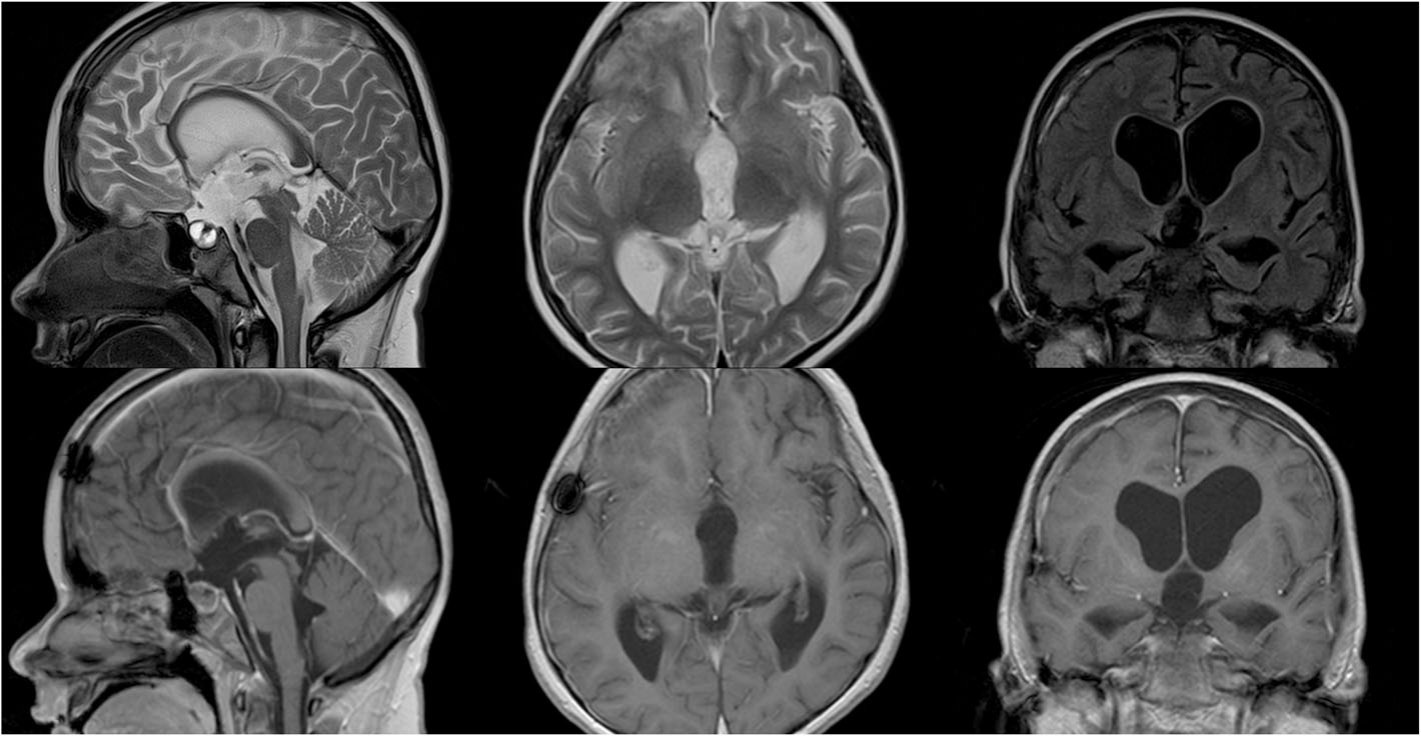
Control magnetic resonance imaging scan (carried out on day 152 after surgery)

A follow-up MRI scan was carried out on day 152 after surgery. The images were compared with those obtained before treatment. Turbo spin echo diffusion-weighted images and dark fluid sequences with T1- and T2-weighted images in sagittal, coronal and transverse views were obtained prior to and after intravenous injection of a paramagnetic. Status after surgical resection of the craniopharyngioma with possible small remnant of solid part of the tumour in sella is represented in Fig. 3.

The patient underwent systematic rehabilitation and monitoring of the treatment for multi-hormonal pituitary insufficiency. Six months after the procedure she still needed substitutional hormone therapy, anti-epileptic treatment and ophthalmic and psychological counselling. A systematic improvement in the patient’s condition was noticeable. Currently, the patient is in good neurological condition without focal damage to the central nervous system. Hormone substitution continues.

## Discussion and conclusions

Craniopharyngiomas (CPs) are tumours that may develop from the sella to the third ventricle and constitute approximately 3% of all intracranial tumours [8]. They are the most common form of non-neuroepithelial neoplasm in paediatric population. The most common location of a craniopharyngioma is the sellar and suprasellar region, with 95% of craniopharyngiomas having a suprasellar component. Its location defines its pathophysiology. There are two different subtypes of CPs that differ clinically and pathologically. Adamantinomatous CP (ACP) which occurs predominantly in the paediatric population and papillary CP (PCP) which is seen mostly among adults. The ACPs are much more common than PCP (9:1) and are pathologically distinct [9]. ACPs are composed of cystic “motor oil-like” component and solid components and frequently contain calcifications that are readily identifiable on neuroimaging. Histologically, they contain nodules of wet keratin, a palisading basal layer of cells, surrounding gliosis, and profuse Rosenthal fibre formation. Craniopharyngiomas can present with hydrocephalus secondary to third ventricle compression [10]. In cases of significant suprasellar extension, non-specific symptoms of intracranial hypertension such as a headache, nausea, and vomiting can also occur [11, 12]. It can compress normal pituitary tissue and result in pituitary deficiencies, particularly of the anterior pituitary hormones. It can also compress the optic chiasm and/or optic nerves and cause different degrees and types of visual disturbances. Cases of isolated oculomotor nerve and abducens nerve palsies have also been described [13]. Chemical meningitis should be considered in a febrile or meningitic patient after the resection of a craniopharyngioma [7]. Chemical meningitis does not necessarily produce classical meningitic symptoms, which makes the diagnosis challenging. Routine follow-up after craniopharyngioma resection typically includes MRI and lumbar puncture. Up to 60–75% of patients with postoperative meningitis have an aseptic form of this condition. However, clinical characteristics and the results of CSF examination in aseptic and bacterial meningitis are quite similar [7, 14, 15].

Aseptic meningitis is caused by various irritants that may trigger inflammation of the meninges. Craniopharyngiomas found in children are usually sellar tumours originating from the remnants of Rathke’s pouch. Although histologically benign, they are considered malignant due to their close proximity to optic nerves, pituitary gland and hypothalamus, and may contribute to visual problems, endocrine deficiency and post-resection obesity [14–16]. Since the chemical environment responsible for inducing aseptic meningitis can also lead to serious cerebrovascular complications, aseptic meningitis and the development of associated complications should remain high on the differential in patients with fever or meningioma after craniopharyngeal resection. The clinical and laboratory picture of aseptic meningitis can be described by: fever of 102 °F (38.9 °C); an absence of CSF rhinorrhoea; a negative CSF culture /gram stain; CSF lactate < 35 mg/dl; CSF WBC < 2000 cells/μl; normal CSF glucosea and a not statistically different CSF protein level [15, 17].

In the presented case, both CT and MRI of the head confirmed the presence of a craniopharyngioma with concomitant active hydrocephalus. Because chemical meningitis rarely occurs after craniopharyngioma resection in children (in our Hospital it is the first case in approximately 20 years), no precautions had been taken. The first 6 days post-surgery were uneventful. It was only the next day that the patient’s status rapidly deteriorated: she presented fever, severe headache, episodes of generalized seizure and progressive disorders of consciousness. Physical examination revealed meningeal signs, including a stiffening of the neck and Kernig’s and Babinski sign on the right side.

Postoperative meningitis still constitutes a diagnostic and therapeutic challenge in patients after craniopharyngioma resection [18]. The result of CSF examination in our patient was similar to those reported by other authors describing aseptic meningitis during the course of craniopharyngioma treatment, either by conventional or endoscopic surgery [7, 15, 18, 19]. The examination of CSF typically demonstrates mild leukocytosis with a left shift, with normal glucose and elevated total protein content [7]. Some authors report elevated levels of cholesterol in the CSF, resulting from a rupture of the suprasellar cyst [20]. Chemical meningitis in craniopharyngioma patients is supposed to be triggered by cholesterol crystals present in the cystic fluid [21, 22]. Authors of several case reports pointed to the rupture of craniopharyngioma and release of its cholesterol-rich content as a cause of aseptic meningitis [21–23]. Another potential causal factor is extravasation of erythrocytes from intraoperatively ruptured blood vessels [24].

Taking into account the rarity and unpredictability of the occurrence of the postoperative complications of the tumour described in the literature, a lack of classic symptoms of meningitis makes it difficult to make a correct diagnosis immediately after the surgery.

A limitation of this study is that only one case is discussed. On the other hand, any well-described report can be very useful to understand pathology, especially when it is rare. The strength of this paper is that a detailed description of a case is provided as well as of the surgical and postoperative procedures undertaken, which can be used as a future guide of how to deal with similar cases.

Chemical meningitis should be considered as a potential early postoperative complication in patients after a craniopharyngioma resection, either conventional or endoscopic. Early diagnosis of this complication requires cooperation with neurosurgeons and the implementation of antibiotic therapy despite a negative result of cerebrospinal fluid examination culture.

## Data Availability

All data generated or analysed during this study are included in this published article.

## Abbreviations

AE1/AE2: Stratified epithelium
anti-CMV: Cytomegalovirus antibodies
anti-EBV: Epstein-Barr virus antibodies
anti-HSV: Herpes simplex virus antibodies
CD 68: Polyclonal antibody CD68
CSF: Cerebrospinal fluid examination
CT: Computed tomography
G1-WHO: The lowest degree of cellular and architectural tumour atypia, according to the World Health Organization
GFAP: Glial fibrillary acidic protein
IgM: Immunoglobulin M
MIB-1: Monoclonal antibody MIB-1
MRI: Magnetic resonance imaging
